# Muscle Strength, Genetic Risk, and Type 2 Diabetes Among Individuals of South Asian Ancestry: A UK Biobank Study

**DOI:** 10.1111/1753-0407.70074

**Published:** 2025-03-27

**Authors:** Ziyuan Chen, Paul James Collings, Mengyao Wang, Haeyoon Jang, Qiaoxin Shi, Hin Sheung Ho, Shan Luo, Shiu Lun Au Yeung, Youngwon Kim

**Affiliations:** ^1^ School of Public Health, the University of Hong Kong Li Ka Shing Faculty of Medicine, Pokfulam Hong Kong China; ^2^ MRC Epidemiology Unit, University of Cambridge School of Clinical Medicine, Institute of Metabolic Science, Cambridge Biomedical Campus Cambridge UK

**Keywords:** genetic susceptibility, muscle strength, south‐Asian ancestry, type 2 diabetes, UK biobank

## Abstract

**Objective:**

To examine the independent and combined associations of genetic risk and muscle strength with the risk of incident type 2 diabetes (T2D) and glycated hemoglobin (HbA1c) among individuals of South Asian ancestry.

**Design and Methods:**

This study included 5288 South Asian individuals (mean age 52.5 years; 52.3% men) from the UK Biobank study. Baseline assessments were conducted between 2006 and 2010. Muscle strength was assessed through a hand dynamometer and expressed relative to fat‐free mass. Sex‐and age‐specific tertiles were used to classify muscle strength into three categories. Genetic risk of T2D was quantified using a weighted polygenic risk score calculated from 22 distinct South Asian‐specific single nucleotide polymorphisms for T2D.

**Results:**

Compared to the bottom tertile of genetic risk for T2D, the highest had increased odds of incident T2D (odds ratio: 1.62; 95% confidence interval [CI]: 1.31–2.00) and HbA1c levels (*β*: 0.80; 95% CI 0.41–1.19). Compared to high muscle strength, low muscle strength was associated with 89% higher odds of incident T2D (odds ratio: 1.89; 95% CI 1.52–2.35) and higher HbA1c levels (*β*: 0.95; 95% CI 0.55–1.35), after adjustment for confounders and genetic susceptibility to T2D. Joint analyses revealed lower muscle strength was consistently associated with higher odds of incident T2D and HbA1c levels across all genetic risk strata.

**Conclusion:**

Polygenic risk scores for T2D could have great prognostic value in preemptively identifying individuals of South Asian ancestry at high genetic risk of T2D. Regardless of T2D genetic risk, greater muscle strength is linked to lower T2D risk and HbA1c levels.

## Graphical abstract

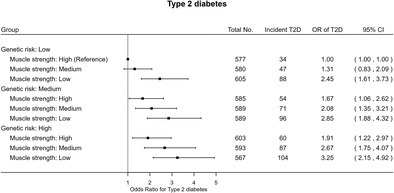

**Summary**

- Polygenic risk scores for type 2 diabetes (T2D) could help in the early identification of South Asian individuals at high genetic risk.
- Higher muscle strength is associated with a lower risk of T2D and reduced HbA1c levels, regardless of genetic susceptibility.
- The consistent inverse relationship between muscle strength and T2D risk, irrespective of genetic traits, indicates that enhancing muscle strength should be a major lifestyle‐modification strategy for T2D prevention among South Asians, including those at elevated genetic risk.

**Abbreviations**
GWAS: genome‐wide association studies
HbA1c: glycated hemoglobin
OR: odds ratio
PRS: polygenic risk score
PRS‐T2D: polygenic risk score for type 2 diabetes
RERI: relative excess risk due to interaction
T2D: type 2 diabetes

## 1 Introduction

Type 2 diabetes (T2D) is recognized as a major global public health crisis affecting more than 500 million individuals worldwide [1]. Evidence indicates that South Asia, the most densely populated region of the world (accounting for approximately a quarter of the world population), has over 120 million diagnosed cases of diabetes. Notably, the epidemic of T2D appears disproportionately prone to South Asians compared to other ethnicities. For example, the prevalence rate of T2D in South Asian countries such as India (9.6%), Pakistan (30.8%), and Bangladesh (14.2%) far surpasses that of European countries (overall 9.2%) [1]. Moreover, South Asians residing in developed countries (the USA, the UK, and Canada) have a much higher prevalence rate of diabetes (ranging from 8.2% to 15.9%) than individuals of European ancestry (ranging from 3.8% to 11.6%) [2, 3]. Over 38 million South Asians are estimated to live in Western countries [4], and these individuals are potentially at a heightened risk of developing T2D (three to four‐fold elevated T2D risk compared to individuals of European ancestry) [5, 6, 7].

Possible explanations for this disparity are multi‐faceted. The onset of diabetes encompasses a complex matrix of non‐modifiable genetic traits and modifiable extrinsic factors that interrelate within a broader framework of physical and sociocultural environments. Studies have highlighted that there is heterogeneity in genetic architecture between individuals of South Asian ancestry and those of European ancestry, particularly in the context of T2D [8, 9]. A genome‐wide association study revealed that South Asians have a higher DNA methylation score across five genetic loci (ABCG1, PHOSPHO1, SOCS3, SREBF1, and TXNIP) for T2D compared with Europeans [10]. Furthermore, evidence suggests a poorer innate insulin secretion and β cell function in non‐diabetic South Asian individuals than in White people [11, 12, 13]. The vast majority of genome‐wide association studies for T2D have been performed with individuals of European ancestry, but multiple novel genetic loci have recently been discovered specifically for individuals of South Asian ancestry [6, 9, 14].

Skeletal muscle, a predominant site of glucose disposal, is a physiological trait determined in part by lifestyle behaviors. Of various risk factors that could influence or regulate glucose metabolism, muscular fitness stands out as a key modifiable target. As such, the role of muscle strength has received growing interest in the prevention and management of diabetes [15, 16]. Research has shown muscle strength to be linked to T2D risk in adult populations [17]. To our understanding, however, no previous research has explored the prospective association of muscle strength with the risk of T2D among South Asians. South Asian individuals tend to possess lower lean muscle mass than individuals of European ancestry [18, 19, 20], suggesting individuals of South Asian ancestry may have lower muscle strength and, consequently, a higher risk of developing T2D. Notably, previous research on the relationship between muscle strength and incident T2D has disregarded the potential influence of genetics. It remains unclear whether the association between muscle strength and T2D incidence is independent of, or possibly varies by, an individual's genetic susceptibility to T2D in individuals of South Asian ancestry. This study aims to explore the independent and combined associations of genetic risk and muscle strength with incident T2D and glycated hemoglobin (HbA1c) levels among individuals of South Asian ancestry.

## 2 Materials and Methods

### 2.1 Study Design and Participants

The current study used data from the UK Biobank, a large‐scale prospective cohort study comprising over half a million UK adults aged 40–69 years at recruitment [21, 22]. Participants were included if they were registered with the National Health Service and lived less than 25 miles from one of 22 designated clinical assessment centers located throughout the UK. The baseline assessment was conducted between March 2006 and October 2010, during which extensive genetic, sociodemographic, behavioral, health, and physical information was collected. This study includes 5228 participants of South Asian ancestry (including participants of Indian (*n* = 4113), Pakistani (*n* = 1061), and Bangladeshi (*n* = 114) origin), who provided complete data for all study variables (Figure S1). Ethnicity was self‐reported and verified by principal component analysis of genetic data. The present analyses excluded prevalent T2D cases, which were defined based on either having self‐reported diabetes (type 1 diabetes, T2D, and gestational diabetes), having diabetes at baseline based on hospital admission records, or having self‐reported diabetes medication use at baseline, following the diabetes adjudication method recommended by the UK Biobank Outcomes and Follow‐up Working Groups [23]. This adjudication strategy for T2D prevalence has shown a 96% sensitivity rate when validated against secondary care data in a subset of UK Biobank participants [24].

### 2.2 Exposures

#### 2.2.1 Genetic Susceptibility for Type 2 Diabetes

Genotyping was performed using the Affymetrix UK Biobank Axiom Array, with imputation of genotypes to a reference panel from the 1000 Genomes Project [22]. Each participant's genetic susceptibility to T2D was quantified by calculating a weighted polygenic risk score (PRS). Initially, 22 genome‐wide significant BMI‐adjusted SNPs (*p* value < 5 × 10^−8^) from a South Asian‐specific GWAS [9] were identified, but 14 uncorrelated, lead SNPs from each locus (in low linkage disequilibrium, *r* ^2^ < 0.001) were included in the PRS calculation. We calculated a weighted continuous PRS variable by summing the products of the number of risk alleles and the corresponding effect estimates at each locus. The calculated PRS variable was normally distributed (Figure S2). Tertiles of PRS were used to classify individuals into low, medium, or high T2D genetic risk groups. The list of SNPs used for the calculation of PRS can be found in Table S1. The PRS for T2D was computed using PLINK 2.0.

#### 2.2.2 Muscle Strength

Handgrip strength, a feasible proxy measure for general muscle strength [25, 26], was measured using a hand dynamometer with a registration capacity of up to 90 kg (Jamar hydraulic J00105). Sitting upright in a chair with the elbow at a 90° angle, participants were asked to squeeze the handle of the dynamometer as strongly as possible for approximately 3 s. Measurements were taken for both hands following the same protocol. To account for variation in lean mass, muscle strength was calculated by dividing the average grip strength from both hands by fat‐free mass [measured by a bioimpedance analyzer (Tanita BC‐418MA)]. This adjustment helps to ensure a more accurate comparison of grip strength regardless of differences in body size [26, 27, 28]. Participants were classified into three age‐and sex‐specific groups (tertiles) of muscle strength.

### 2.3 Outcomes

#### 2.3.1 Glycated Hemoglobin (HbA1c)

HbA1c was included as the outcome in the cross‐sectional analyses. HbA1c (measured in mmol/mol) was ascertained from biological samples collected at baseline (2006–2010) and analyzed using the Bio‐Rad Variant II Turbo Hemoglobin Testing System. Full details of the procedure are provided elsewhere [29].

#### 2.3.2 Diabetes Ascertainment

Incident T2D was defined according to the International Classification of Diseases code (ICD‐10: E11) based on hospital admission and mortality records (accrued until December 5th 2022 for participants in England and Wales, and until December 18th 2022 for participants in Scotland).

### 2.4 Confounders

We considered the following variables to be potential confounders of the associations between muscle strength and outcomes: sex (male/female), age (years), smoking status (never, former, current), alcohol consumption (never, previous, currently less than 3 times/week, currently ≥ 3 times/week), employment status (unemployed, employed), Townsend Deprivation Index (an integrated score based on postcode that takes into account employment, car ownership, home ownership, and household overcrowding; higher scores indicate a greater level of deprivation), dietary habits (red meat consumption, vegetable intake, fish intake, fruit intake, frequency of adding salt to food after cooking), moderate‐to‐vigorous physical activity (minutes/day), and body fat percentage.

### 2.5 Statistical Analysis

Given that the exact date of T2D onset was not directly measured in UK Biobank, diagnosis dates were estimated rather than precisely recorded. Specifically, dates of diagnosis were not always available in hospital admission records. Instead, the diagnosis date may have been inferred by taking the midpoint between the last record without diabetes and the first record with evidence of diabetes, such as diabetes medications being prescribed or any activities related to diabetes care. To minimize the bias from diabetes developing some time prior to the estimated date based on hospital admission records, logistic regression models were used to examine the associations between muscle strength and PRS with incident T2D, following the methodology of previous research [30]. To examine the shape of associations between PRS values and muscle strength with incident T2D, cubic spline models with five equidistant knots were utilized (Figures S3 and S4). We fit three models with sequential adjustment for potential confounders. In Model 1, using muscle strength as the main exposure, no adjustment was made. In Model 2, we accounted for potential confounders. In Model 3, an additional adjustment was made for PRS and the first 20 principal components of ancestry (to correct for population stratification). In models using PRS as the exposure variable, we adjusted for age, sex, and the first 20 principal components of ancestry. Linear regression models were fit to estimate the cross‐sectional associations between PRS‐T2D and muscle strength with HbA1c. Models were sequentially adjusted for all the aforementioned confounders. Interactions (multiplicative and additive interaction [RERI] [31]) between the three categories of PRS and tertiles of muscle strength in relation to incident T2D were tested with the adjustment for all confounders. To explore whether muscle strength could modify the genetic risk of developing T2D, we constructed joint models where a total of nine discrete genotype‐muscle strength groups (e.g., three PRS categories and three muscle strength categories combined) were used. Three sensitivity analyses were conducted to assess the robustness of the findings. First, we performed time‐to‐event analyses (i.e., Cox proportional hazard models with age as the underlying timescale) on the associations of genetic susceptibility to T2D and muscle strength with incident T2D (Figure S5 and Table S2). Second, we conducted analyses after standardizing muscle strength (fat free mass adjusted) for age in sex‐specific linear regression models, calculating the residuals for each participant, and then categorizing the derived muscle strength variable into three groups based on tertiles of the residuals while taking into account the age and sex characteristics of the participants (Figure S6 and Table S3). Additionally, we performed an analysis that included individuals with pre‐existing diabetes to evaluate how this inclusion affects the magnitude of the relationship between muscle strength and HbA1c levels (Table S4). All statistical analyses were performed using Stata/MP version 17.0 software (StataCorp LLC).

## 3 Results

The primary analysis included 5288 South Asian individuals (52% male; average age at recruitment 52.5 years [SD 8.4]); no statistically significant difference was observed for PRS‐T2D across three muscle strength groups (one‐way ANOVA test, *p* = 0.34). Most participants were not former or current smokers (80.1%), and participants had a grip strength of 27.4 kg on average. Table S5 presents an overview of participant characteristics, overall and stratified by muscle strength tertiles.

Table 1 shows the odds of T2D as a function of PRS values and muscle strength, separately. Compared to the low genetic risk group, medium (OR 1.35, 95% CI: 1.09–1.68) and high (OR 1.62, 95% CI: 1.31–2.00) T2D genetic risk was associated with 35% and 62% higher odds of T2D, respectively, after adjusting for age, sex, and the first 20 principal components. The medium (OR 1.43, 95% CI: 1.15–1.80) and low muscle strength (OR 2.13, 95% CI: 1.73–2.63) tertiles had 43% and over 2.13‐fold increases in the odds of T2D, respectively, compared to the highest tertile of muscle strength (Model 1). This association remained strong after adjusting for potential confounders and PRS‐T2D (Model 3). Similar associations between genetic susceptibility to T2D, muscle strength, and incident T2D were also observed in the sensitivity analyses (Tables S2 and S3). Table 2 presents the associations of PRS for T2D and muscle strength with HbA1c. Higher PRS‐T2D was associated with higher levels of HbA1c, and higher HbA1c levels were observed among individuals with the lowest level of muscle strength after adjustment for confounders.

**TABLE 1:** Associations of polygenic risk score and muscle strength with incident type 2 diabetes.

T2D outcome				Odds ratio of type 2 diabetes (95% confidence intervals)		
	Comparisons	No. of participants	No. of cases	Model 1	Model 2	Model 3
		5288	641			
Tertiles of polygenic risk score for T2D						
	Low (reference)	1762	169	Reference		
	Middle	1763	221	1.35 (1.09–1.68)		
	High	1763	251	1.62 (1.31–2.00)		
Tertiles of muscle strength						
	High (reference)	1765	148	Reference	Reference	Reference
	Middle	1762	205	1.43 (1.15–1.80)	1.34 (1.06–1.68)	1.32 (1.05–1.66)
	Low	1761	288	2.13 (1.73–2.63)	1.88 (1.51–2.33)	1.89 (1.52–2.35)

*Note:* Model 1: adjusted for age, sex and the first 20 principal components of genetic ancestry in models for polygenic risk scores and no confounders in models for muscle strength; Model 2: In models for muscle strength we adjusted for age, sex, smoking status (never, previous, current), employment (unemployed, employed), Townsend Deprivation Index, alcohol consumption (never, previous, currently < 3 times/week, currently > = 3 times/week), dietary factors (red meat consumption, vegetable intake, fruit intake, fish intake, frequency of adding salt to food after cooking), moderate to vigorous physical activity (min/day), and body fat percentage; Model 3: adjusted for all confounders in model 2 with an additional adjustment for T2D polygenic risk score and the first 20 principal components of genetic variant.

**TABLE 2:** Associations of polygenic risk score for type 2 diabetes and muscle strength with glycated hemoglobin.

HbA1c outcome			Beta coefficient of glycated hemoglobin (95% confidence intervals)		
	Comparisons	No. of participants	Model 1	Model 2	Model 3
		5288			
Tertiles of polygenic risk score for T2D					
	Low (reference)	1762	Reference		
	Middle	1763	0.58 (0.21, 0.96)		
	High	1763	0.80 (0.41, 1.19)		
Tertiles of muscle strength					
	High (reference)	1765	Reference	Reference	Reference
	Middle	1762	0.70 (0.30–1.09)	0.56 (0.17–0.95)	0.59 (0.20–0.98)
	Low	1761	1.16 (0.76–1.55)	0.92 (0.53–1.32)	0.95 (0.55–1.35)

*Note:* Model 1: adjusted for age, sex and the first 20 principal components of genetic ancestry in models for polygenic risk scores and no confounders in models for muscle strength; Model 2: In models for muscle strength we adjusted for age, sex, smoking status (never, previous, current), employment (unemployed, employed), Townsend Deprivation Index, alcohol consumption (never, previous, currently < 3 times/week, currently > = 3 times/week), dietary factors (red meat consumption, vegetable intake, fruit intake, fish intake, frequency of adding salt to food after cooking), moderate to vigorous physical activity (min/day) and body fat percentage; Model 3: adjusted for all confounders in model 2 with an additional adjustment for T2D polygenic risk score and first 20 principal components of genetic variant.

**FIGURE 1:**
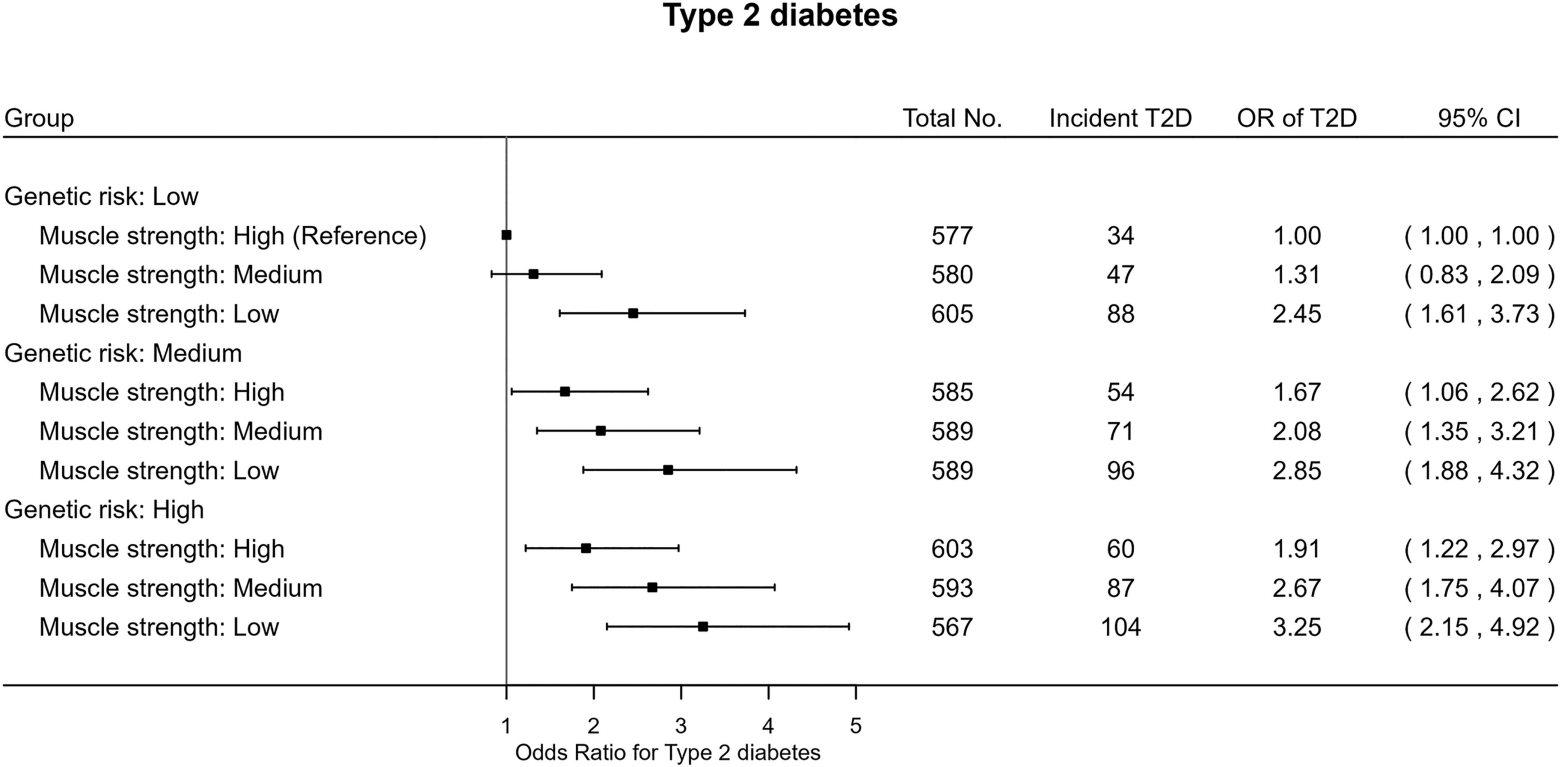
Joint associations of genetic predisposition and muscle strength with incident type 2 diabetes. Logistic regression model was employed adjusting for age, sex, smoking status (never, previous, current), employment (unemployed, employed), Townsend Deprivation Index, alcohol consumption (never, previous, currently < 3 times/week, currently > = 3 times/week), dietary factors (red meat consumption, vegetable intake, fruit intake, fish intake, frequency of adding salt to food after cooking), moderate to vigorous physical activity (min/day), body fat percentage, and the first 20 principal components of genetic variant. Abbreviations: OR, odds ratio; T2D, type 2 diabetes; CI, confidence interval. Multiplicative interaction (*p* = 0.16) and additive interaction (*p* = 0.68).

**FIGURE 2:**
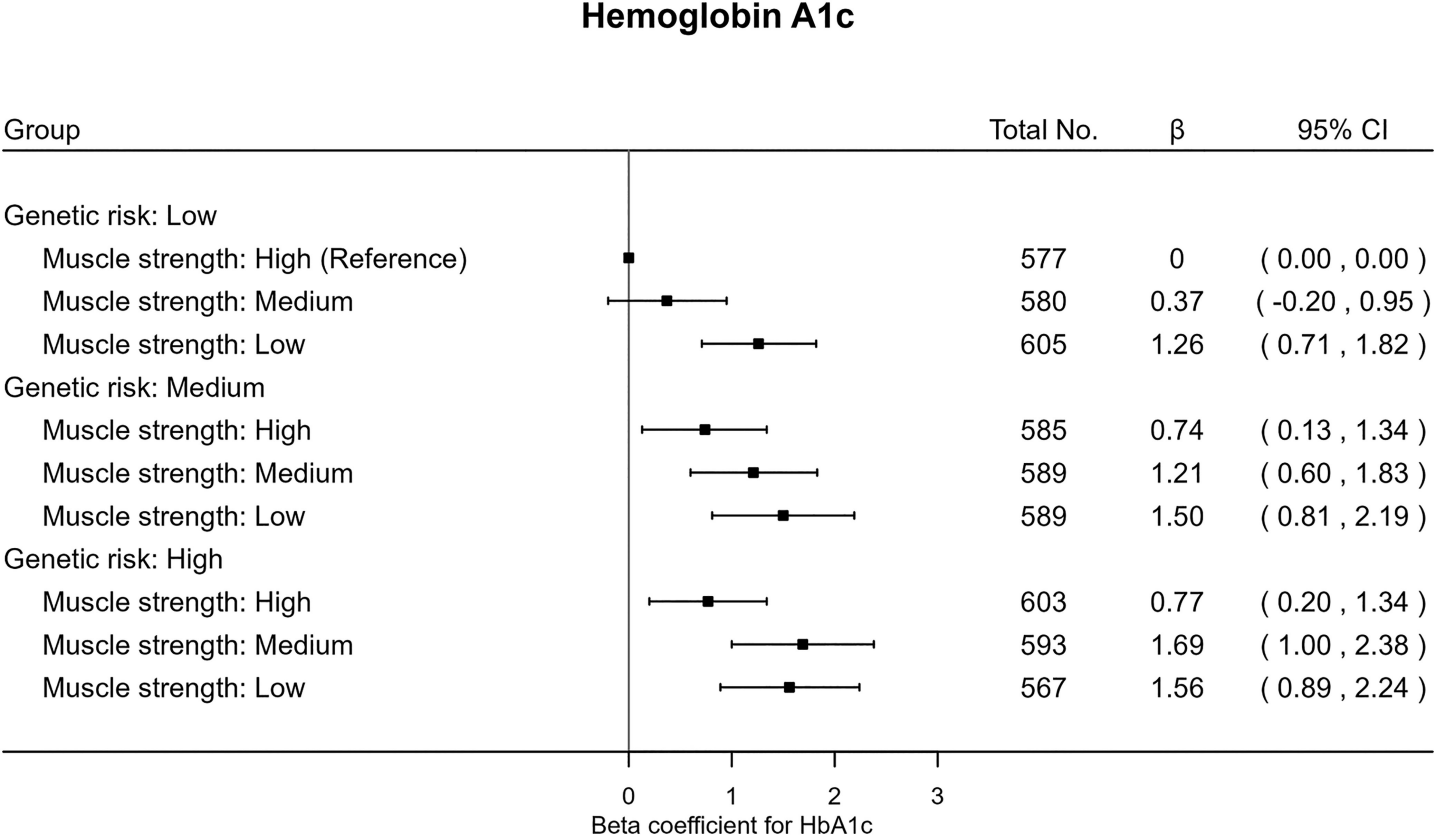
Joint associations of genetic predisposition and muscle strength with levels of hemoglobin A1c. General linear regression model was employed adjusting for age, sex, smoking status (never, previous, current), employment (unemployed, employed), Townsend Deprivation Index, alcohol consumption (never, previous, currently < 3 times/week, currently > = 3 times/week), dietary factors (red meat consumption, vegetable intake, fruit intake, fish intake, frequency of adding salt to food after cooking), moderate to vigorous physical activity (min/day), body fat percentage, and the first 20 principal components of genetic variant. Abbreviations: β, beta coefficient; CI, confidence interval. Multiplicative interaction (*p* = 0.30) and additive interaction (*p* = 0.91).

The joint analysis (Figure 1) revealed that, within each genetic risk group, low muscle strength had relatively higher odds of T2D compared with high muscle strength. Individuals with high T2D genetic susceptibility and low muscle strength had over three times higher odds (OR 3.25 95% [CI: 2.15–4.92]) of T2D compared with the reference category of low genetic risk and high muscle strength. Similar patterns were found in the joint association of combined categories of genetic risk for T2D and muscle strength with HbA1c (Figure 2). There was no strong evidence of multiplicative (*p* = 0.16) or additive interaction (*p* = 0.68) between muscle strength and genetic risk relative to incident T2D.

## 4 Discussion

This is the first study exploring the associations of genetic predisposition to T2D and muscle strength with incident T2D and HbA1c in any South Asian population, an underrepresented sub‐population group in the current evidence base. South Asian individuals with higher genetic susceptibility to T2D exhibited higher levels of HbA1c and elevated odds of T2D. Low muscle strength was strongly linked to T2D risk in this specific ethnic group, independently of genetic susceptibility to T2D. Moreover, across all strata of T2D genetic risk, individuals with lower muscle strength had higher T2D risk and HbA1c levels: no evidence of multiplicative or additive interactions.

These findings address the gap in the current literature and go beyond previous investigations by further incorporating each individual's genetic make‐up in exploring the role of muscle strength in relation to T2D incident among individuals of South Asian ancestry. Our findings expand upon the existing evidence on the inverse association between muscle strength and incident T2D [32, 33]. A recent study using participants from UK Biobank data found that the lowest quintile of relative grip strength (kg per kg of body weight) had more than 2‐fold increased hazard of developing T2D compared to the highest quintile, after adjustment for potential confounders [32]. Another investigation involving data of six ethnic groups (Dutch, South Asian, African, Ghanaian, Turkish and Moroccan) suggested that the varying levels of grip strength across ethnic groups did not account for the variations in the T2D prevalence for the respective ethnicities [33]. This observation poses a hypothesis that genetic factors, alongside muscle strength, may contribute to the varying levels of susceptibility to T2D among individuals of different ancestries. Nonetheless, no previous research [32, 33, 34, 35] took into account individuals' unique genetic architecture.

The joint models yielded unique insights into the relationship between increased muscle strength and the onset of T2D across levels of T2D genetic susceptibility. Our results indicated that South Asian individuals with low muscle strength at high genetic risk of T2D exhibited the highest risk of T2D compared to the combined category of low genetic risk and high muscle strength. In addition, we found lower muscle strength to be consistently associated with higher T2D risk compared to higher muscle strength across all genetic risk strata, while there was no evidence of interaction. These findings suggest that improving muscle strength could serve as a key behavioral target for the prevention of T2D among individuals of South Asian ancestry, regardless of their genetic risk of T2D.

Prior research has explored the potential benefits of muscle strength in the prevention of common chronic diseases primarily in populations of European descent [26, 36, 37], the present study provides novel insights into the combined associations of genetic risk and muscle strength levels for T2D prevention among individuals of South Asian ancestry. Previous intervention studies have demonstrated the beneficial effects of resistance exercise or muscle‐strengthening activities on glycemic control, including reductions in HbA1c in individuals with T2D [38, 39]. A recent trial involving South Asian individuals with T2D revealed that engaging in resistance exercise for 2 days per week resulted in a significant absolute reduction in HbA1c levels [40].

To the best of our knowledge, however, there has been no experimental and epidemiological research that takes into account individuals' inherited genetic risk of T2D in exploring the associations of muscle strength with T2D incidence among individuals of South Asian ancestry. Clinical trials using individuals' genetic susceptibility are essential to establish causal evidence on whether heightened genetic risk of T2D can be mitigated through improving muscle strength. The findings from our study could offer valuable benchmarks for future precision medicine initiatives where T2D prevention strategies could be developed and targeted towards individuals of South Asian ancestry with high genetic susceptibility to T2D.

## 5 Strengths and Limitations

The present study was conducted in individuals of South Asian ancestry—an understudied and yet at‐risk group for diabetes. We computed PRS for T2D using SNPs obtained from the most recent GWAS of a South Asian sample. There are several limitations to this study. The associations reported in this study do not imply causation, as it is an observational study. Some incident T2D cases that would have been diagnosed based on primary care data might not have been adjudicated, given that we only used secondary care and mortality records. Findings of our study may not be generalizable to individuals of South Asian ancestry residing in other countries as well as other ethnic groups. Moreover, although we made adjustments for multiple potential confounding factors, there may still be residual confounding as a result of measurement error and unmeasured confounders.

## 6 Conclusion

Polygenic risk scores for T2D could have great prognostic value in preemptively identifying individuals of South Asian ancestry whose risk of developing T2D is high. Individuals of South Asian descent with higher levels of muscle strength exhibit a reduced risk of T2D and lower HbA1c levels, regardless of their genetic susceptibility. The consistent inverse associations of muscle strength with T2D risk across varying levels of genetic risk suggest that improving muscle strength should be a key behavioral target for the prevention of T2D for all individuals of South Asian ancestry, including those at high genetic risk. Further research is warranted to determine whether the associations are causal.

## Author Contributions

Z.C. and Y.K. initialized the conceptualization and design of this study, developed the analysis plans, and defined the key study variables. Z.C. curated the data, performed all statistical analyses, interpreted the results, and wrote the first draft of the manuscript. Y.K. secured funding for the conduct of this study, contributed to data curation, provided substantial edits and critical reviews, and led the statistical analyses and study administration. All authors critically reviewed the manuscript, approved the final version, and agreed to be responsible for all facets of this work.

## Ethics Statement

The UK Biobank has obtained ethical approval from the NHS National Research Ethics Service, ensuring adherence to ethical guidelines. All participants provided written informed consent prior to their involvement in the study.

## Consent

The authors have nothing to report.

## Conflicts of Interest

The authors declare no conflicts of interest.

## Supporting information


Data S1.

## Data Availability

The study's findings are based on data from the UK Biobank. Access to this data is restricted and was obtained under a specific license for the current study. Interested parties may contact the authors to request the data, subject to UK Biobank's approval.
