# Supplementary material for: Muscle Strength, Genetic Risk, and Type 2 Diabetes Among Individuals of South Asian Ancestry: A UK Biobank Study
Source: J Diabetes. 2025 Mar 27;17(4):e70074. doi: 10.1111/1753-0407.70074 (PMC11950151; doi:10.1111/1753-0407.70074)
Supplement: Supplementary file 1 — Data S1. [file JDB-17-e70074-s001.docx]

**Supplement Material**

**Supplemental Table S1.** Lists of Single-Nucleotide Polymorphisms (SNPs) related to type 2 diabetes in South Asian population.

**Supplemental Tables S2-S4.** Results from sensitivity analyses.

**Supplemental Table S5.** Participant characteristics overall and within tertiles of muscle strength

**Figure S1**. Figure 1. Participants’ flow chart

**Figure S2.** Distribution of polygenic risk scores for type 2 diabetes.

**Figure S3**. Cubic spline analysis of the association between polygenic risk score and incident type 2 diabetes

**Figure S4**. Cubic spline analysis of the association between relative muscle strength and incident type 2 diabetes

**Figures S5-S6.** Results from sensitivity analyses.

Table S1. A list of 14 BMI-adjusted Single-Nucleotide Polymorphism (SNPs) known to be associated with type 2 diabetes for individuals of South Asian ancestry with genome-wide significance at a p-value of 5×10^-8^ and in low linkage disequilibrium defined according to r^2^<0.001.

| SNP | Effect allele frequency | Effect allele | Other allele | Beta | P-value |
| --- | --- | --- | --- | --- | --- |
| rs13389219 | 0.7439 | C | T | 0.098 | 2.69E-08 |
| rs2138157 | 0.7624 | C | A | 0.103 | 1.40E-08 |
| rs2012444 | 0.8813 | C | T | 0.147 | 5.78E-10 |
| rs2877716 | 0.7738 | C | T | 0.112 | 1.09E-09 |
| rs6780171 | 0.4252 | A | T | 0.132 | 7.57E-18 |
| rs7766070 | 0.2663 | A | C | 0.123 | 4.79E-13 |
| rs35859536 | 0.7631 | C | T | 0.112 | 4.63E-10 |
| rs10811661 | 0.8628 | T | C | 0.166 | 2.59E-13 |
| rs12780155 | 0.1758 | A | T | 0.139 | 2.61E-12 |
| rs2497306 | 0.3411 | C | A | 0.109 | 8.63E-12 |
| rs7903146 | 0.3095 | T | C | 0.264 | 7.94E-59 |
| rs234866 | 0.6914 | G | A | 0.105 | 1.79E-09 |
| rs10908278 | 0.4161 | T | A | 0.088 | 3.71E-08 |
| rs13039863 | 0.2457 | C | G | 0.121 | 1.37E-11 |

Supplementary Table S2. Associations of polygenic risk score and muscle strength with incident type 2 diabetes using Cox regression models

| T2D |  |  |  | Hazard Ratio of Type 2 Diabetes (95% confidence intervals) | | |
| --- | --- | --- | --- | --- | --- | --- |
| Outcome | Comparisons | No. of Participants | No. of cases | Model 1 | Model 2 | Model 3 |
|  |  | 5288 | 641 |  |  |  |
|  | Tertiles of polygenic |  |  |  |  |  |
|  | risk score for T2D |  |  |  |  |  |
|  | Low (Reference) | 1762 | 169 | Reference |  |  |
|  |  |  |  |  |  |  |
|  | Middle | 1763 | 221 | 1.32 (1.08-1.61) |  |  |
|  | High | 1763 | 251 | 1.56 (1.28-1.90) |  |  |
|  | Tertiles of muscle strength |  |  |  |  |  |
|  | High (Reference) | 1765 | 148 | Reference | Reference | Reference |
|  |  |  |  |  |  |  |
|  | Middle | 1762 | 205 | 1.42 (1.16-1.76) | 1.35 (1.09-1.67) | 1.33 (1.08-1.66) |
|  | Low | 1761 | 288 | 2.06 (1.69-2.52) | 1.87 (1.53-2.28) | 1.87 (1.53-2.30) |

Note:

Cox regression models with age as the underlying timescale.

Model 1: adjusted for sex and the first 20 principal components of genetic ancestry in models for polygenic risk scores and no confounders in models for muscle strength.

Model 2: In models for muscle strength we adjusted for sex, smoking status (never, previous, current), employment (unemployed, employed), Townsend Deprivation Index, alcohol consumption (never, previous, currently <3 times/week, currently >=3 times/week), dietary factors (red meat consumption, vegetable intake, fruit intake, salt intake, fish intake), moderate to vigorous physical activity (min/d), and body fat percentage.

Model 3: adjusted for all confounders in model 2 with an additional adjustment for T2D polygenic risk score and the first 20 principal components of genetic variant.

Supplementary Table S3. Associations of muscle strength with incident type 2 diabetes using logistic regression models using residuals-defined muscle strength categories

| T2D |  |  |  | Odds Ratio of Type 2 Diabetes (95% confidence intervals) | | |
| --- | --- | --- | --- | --- | --- | --- |
| Outcome | Comparisons | No. of Participants | No. of cases | Model 1 | Model 2 | Model 3 |
|  | Tertiles of muscle strength |  |  |  |  |  |
|  | High (Reference) | 1763 | 143 | Reference | Reference | Reference |
|  |  |  |  |  |  |  |
|  | Middle | 1763 | 205 | 1.49 (1.19-1.87) | 1.39 (1.10-1.74) | 1.37 (1.09-1.72) |
|  | Low | 1762 | 293 | 2.26 (1.83-2.79) | 2.01 (1.62-2.50) | 2.02 (1.62-2.51) |

Note:

Model 1: adjusted for age, sex and the first 20 principal components of genetic ancestry in models for polygenic risk scores and no confounders in models for muscle strength.

Model 2: In models for muscle strength we adjusted for age, sex, smoking status (never, previous, current), employment (unemployed, employed), Townsend Deprivation Index, alcohol consumption (never, previous, currently <3 times/week, currently >=3 times/week), dietary factors (red meat consumption, vegetable intake, fruit intake, salt intake, fish intake), moderate to vigorous physical activity (min/d), and body fat percentage.

Model 3: adjusted for all confounders in model 2 with an additional adjustment for T2D polygenic risk score and the first 20 principal components of genetic variant.

Table S4. Associations of polygenic risk score for type 2 diabetes and muscle strength with glycated hemoglobin without excluding individuals with pre-existing diabetes. (n=6,362)

| HbA1c |  |  | Beta Coefficient of Glycated hemoglobin (95% confidence intervals) | | |
| --- | --- | --- | --- | --- | --- |
| Outcome | Comparisons | No. of Participants | Model 1 | Model 2 | Model 3 |
|  |  | 6,362 |  |  |  |
|  | Tertiles of polygenic |  |  |  |  |
|  | risk score for T2D |  |  |  |  |
|  | Low (Reference) | 2,120 | Reference |  |  |
|  |  |  |  |  |  |
|  | Middle | 2,121 | 1.25 (0.66-1.85) |  |  |
|  | High | 2,121 | 2.21 (1.59-2.83) |  |  |
|  | Tertiles of muscle strength |  |  |  |  |
|  | High (Reference) | 2,123 | Reference | Reference | Reference |
|  |  |  |  |  |  |
|  | Middle | 2,120 | 1.60 (1.03-2.18) | 1.30 (0.74-1.86) | 1.35 (0.79-1.90) |
|  | Low | 2,119 | 3.50 (2.86-4.15) | 2.81 (2.19-3.44) | 2.86 (2.24-3.49) |

Note:

Model 1: adjusted for age, sex and the first 20 principal components of genetic ancestry in models for polygenic risk scores and no confounders in models for muscle strength.

Model 2: In models for muscle strength we adjusted for age, sex, smoking status (never, previous, current), employment (unemployed, employed), Townsend Deprivation Index, alcohol consumption (never, previous, currently <3 times/week, currently >=3 times/week), dietary factors (red meat consumption, vegetable intake, fruit intake, fish intake, frequency of adding salt to food after cooking), moderate to vigorous physical activity (min/day) and body fat percentage.

Model 3: adjusted for all confounders in model 2 with an additional adjustment for T2D polygenic risk score and first 20 principal components of genetic variant.

Table S5. Participant characteristics overall and within tertiles of muscle strength

|  |  | Tertiles of muscle strength | | | |
| --- | --- | --- | --- | --- | --- |
| Variables | All  (N= 5,288) | Low  (N= 1,761) | Middle  (N= 1,762) | High  (N= 1,765) | *P* _group-difference_ |
| Age | 52.5 (8.4) | 52.7 (8.4) | 52.5 (8.4) | 52.3 (8.3) | 0.39 |
| Sex |  |  |  |  | 1.00 |
| Men | 2,766 (52.3) | 921 (52.3) | 922 (52.3) | 923 (52.3) |  |
| Women | 2,522 (47.7) | 840 (47.7) | 840 (47.7) | 842 (47.7) | <0.01 |
| T2D incident cases | 641 | 288 | 205 | 148 |  |
| Smoking status |  |  |  |  | 0.36 |
| Never | 4,233 (80.1) | 1,422 (80.8) | 1,419 (80.5) | 1,3922 (78.9) |  |
| Previous | 607 (11.5) | 200 (11.4) | 203 (11.5) | 204 (11.6) |  |
| Current | 448 (8.5) | 139 (7.9) | 140 (8.0) | 169 (9.6) |  |
| Employment |  |  |  |  | <0.01 |
| Unemployed | 1,670 (31.6) | 664 (37.7) | 520 (29.5) | 486 (27.5) |  |
| Employed | 3,618 (68.4) | 1097 (62.3) | 1,242 (70.5) | 1,279 (72.5) |  |
| Townsend Deprivation Index | -0.03 (3.1) | 0.4 (3.0) | 0.1 (3.0) | -0.6 (3.0) | <0.01 |
| Alcohol consumption |  |  |  |  | <0.01 |
| Never | 2,010 (38.0) | 742 (42.1) | 687 (39.0) | 581 (32.9) |  |
| Previous  Current (<3 times/week) | 279 (5.3)  2,119 (40.1) | 115 (6.5)  660 (37.5) | 92 (5.2)  710 (40.3) | 72 (4.1)  749 (42.4) |  |
| Current (≥3 times/week) | 880 (16.6) | 244 (13.9) | 273 (15.5) | 363 (20.6) |  |
| Red meat intake (days/week) | 0.6 (0.6) | 0.6 (0.6) | 0.5 (0.6) | 0.6 (0.6) | 0.52 |
| Fruit and vegetable intake (servings/day) | 1.9 (1.2) | 1.9 (1.3) | 1.9 (1.2) | 1.9 (1.2) | 0.49 |
| Additional salt to food after cooking  Never/Rarely  Sometimes  Usually  Always | 2,109 (39.9)  1,817 (34.4)  762 (14.4)  600 (11.4) | 664 (37.7)  625 (35.5)  242 (13.7)  230 (13.1) | 705 (40.0)  595 (33.8)  261 (13.8)  201 (11.4) | 740 (41.9)  597 (33.8)  259 (14.7)  169 (9.6) | 0.02 |
| Fish intake  Never  Less than once a week  Once a week  More than once a week | 1,726 (32.6)  1,574 (29.8)  1,439 (27.2)  549 (10.4) | 646 (36.7)  476 (27.0)  478 (27.1)  161 (9.1) | 590 (33.5)  530 (30.1)  461 (26.2)  181 (10.3) | 490 (27.8)  568 (32.2)  500 (28.3)  207 (11.8) | <0.01 |
| Moderate to vigorous physical activity (min/d) | 49.2 (65.1) | 47.3 (64.9) | 47.5 (62.5) | 52.8 (67.7) | 0.02 |
| Polygenic risk score for T2D | 1.9 (0.3) | 1.9 (0.3) | 1.9 (0.3) | 1.9 (0.3) | 0.34 |
| Fat-free mass (kg) | 49.6 (10.2) | 51.0 (10.7) | 49.6 (10.1) | 48.1 (9.7) | <0.01 |
| muscle strength (kg) | 27.4 (10.6) | 20.4 (8.3) | 28.7 (8.6) | 34.2 (9.9) | <0.01 |
| muscle strength/fat-free mass (kg) | 0.5 (0.2) | 0.4 (0.1) | 0.5 (0.1) | 0.7 (0.1) | <0.01 |
| Glycated hemoglobin (mmol/mol) | 37.5 (5.9) | 38.0 (6.2) | 37.6 (6.1) | 36.9 (5.4) | <0.01 |

Note: Continuous variables are reported as means with corresponding standard deviations. Categorical variables are presented as numbers with percentages. One-way Analysis of Variance (ANOVA) and Chi-Square test were used to compare the differences in the continuous variables and categorical variables, respectively, across the three muscle strength groups.


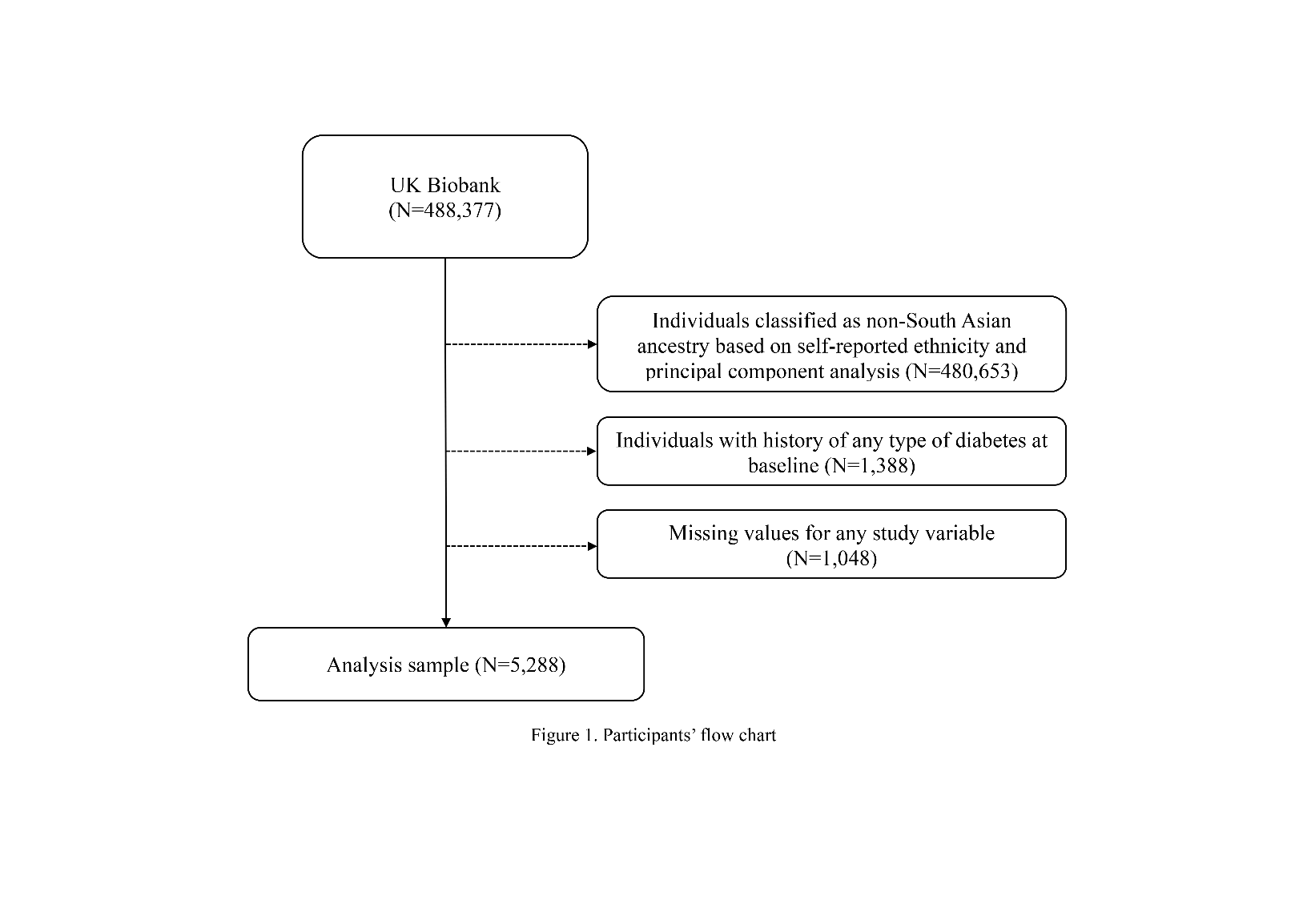


Figure S1. Participants’ flow chart


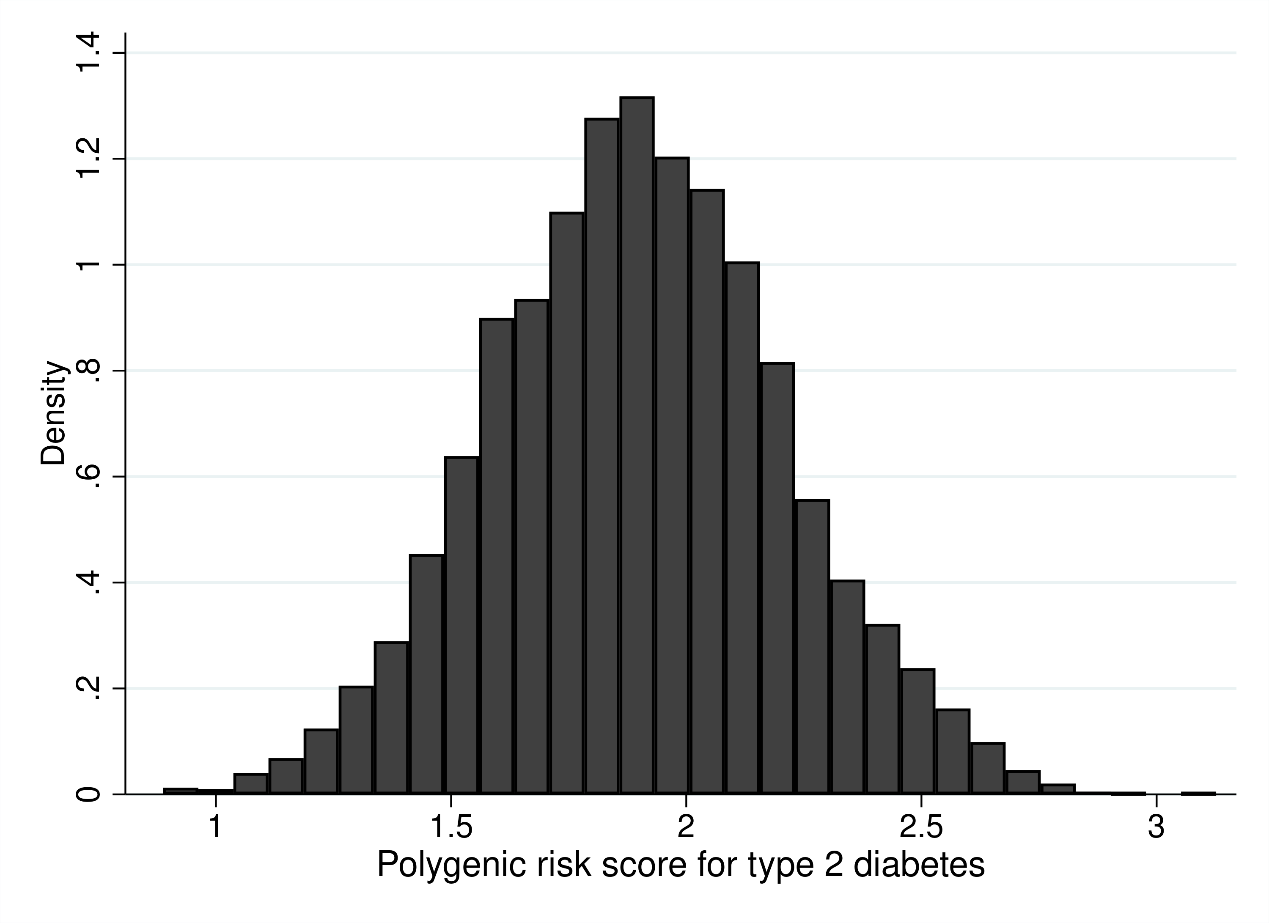


Figure S2. Distribution of polygenic risk score (PRS) for type 2 diabetes using 14 uncorrelated SNPs (genome-wide significant at P <5×10^-8^ and in low linkage disequilibrium defined according to r^2^<0.001).


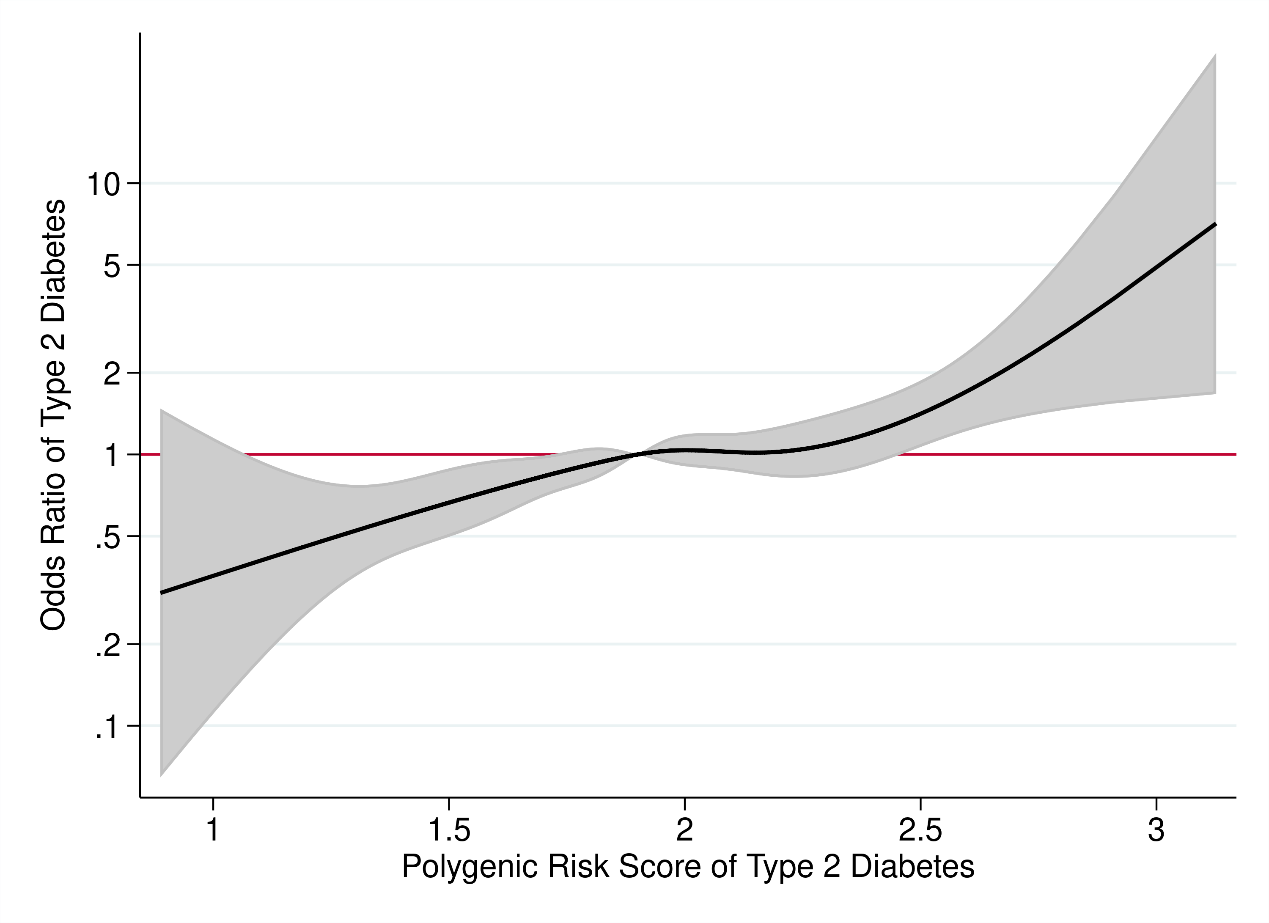


Figure S3. Cubic spline analysis of the association between polygenic risk score and incident type 2 diabetes

Note: The first 20 principal components of ancestry were adjusted to correct for population stratification. Results are expressed relative to PRS=1.9 (sample mean). Abbreviations: Odds, odds ratio


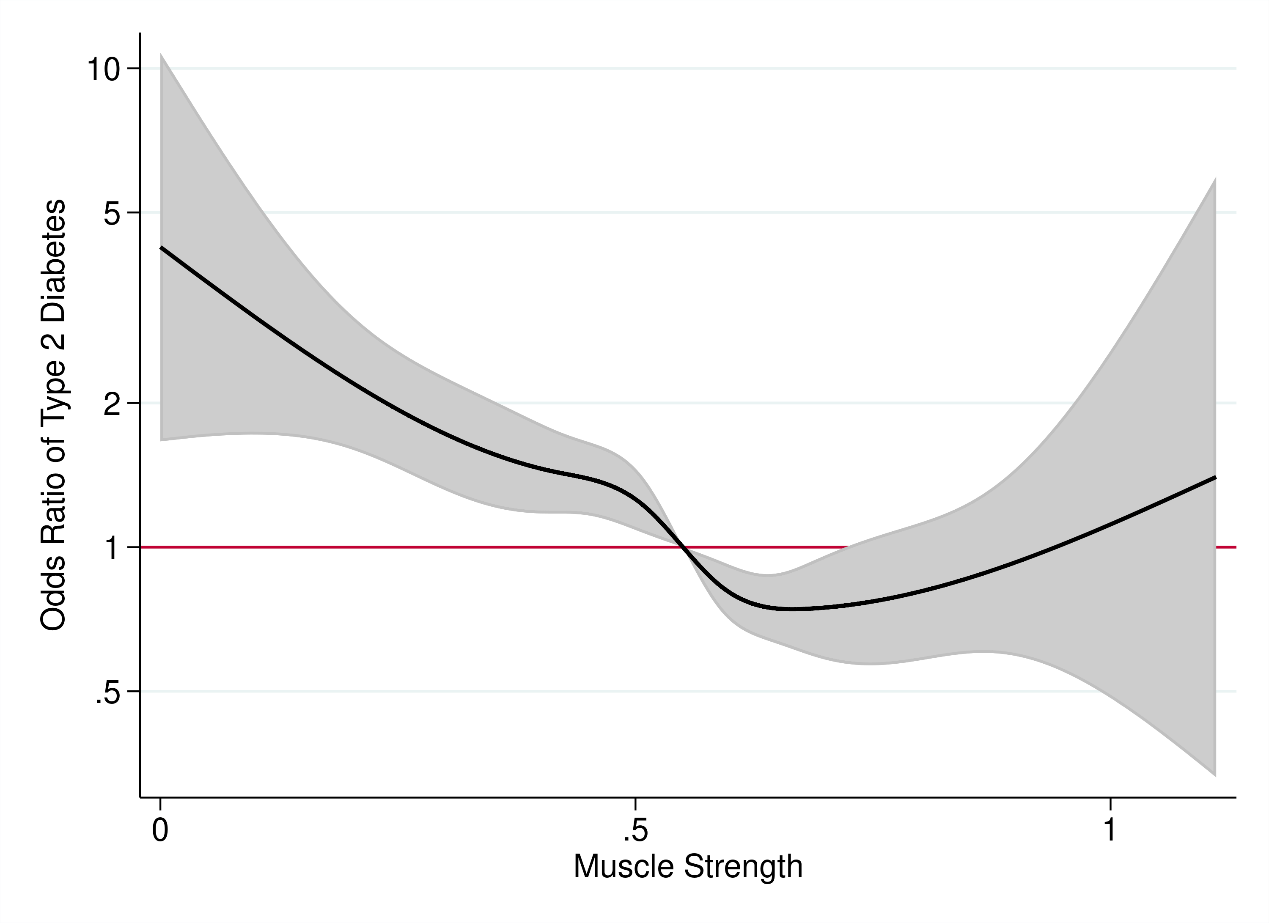


Figure S4. Cubic spline analysis of the association between relative muscle strength and incident type 2 diabetes

Note: Adjustments were made for age, sex, smoking status (never, previous, current), employment (unemployed, employed), Townsend Deprivation Index, alcohol consumption (never, previous, currently <3 times/week, currently >=3 times/week), dietary factors (red meat consumption, vegetable intake, fruit intake, fish intake, frequency of adding salt to food after cooking), moderate to vigorous physical activity (min/d), PRS to T2D and the first 20 principal components of genetic variant . Results are expressed relative to muscle strength (fat free mass adjusted) = 0.55 (sample mean). Abbreviations: Odds, odds ratio


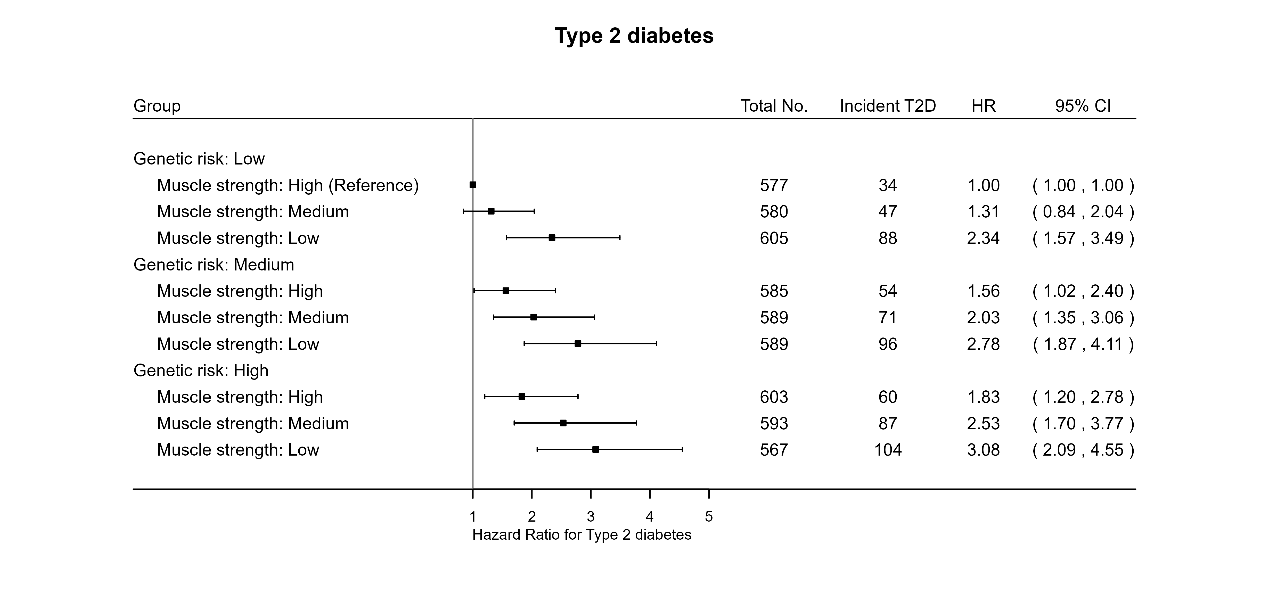


Figure S5. Joint associations of muscle strength, T2D genetic risk with incident T2D.

Cox regression models using age as the underlying timescale were adjusted for sex, smoking status (never, previous, current), employment (unemployed, employed), Townsend Deprivation Index, alcohol consumption (never, previous, currently <3 times/week, currently >=3 times/week), dietary factors (red meat consumption, vegetable intake, fruit intake, salt intake, fish intake), and moderate to vigorous physical activity (min/d), and the first 20 principal components of genetic variant. Abbreviations: T2D, type 2 diabetes. HR, hazard ratio.


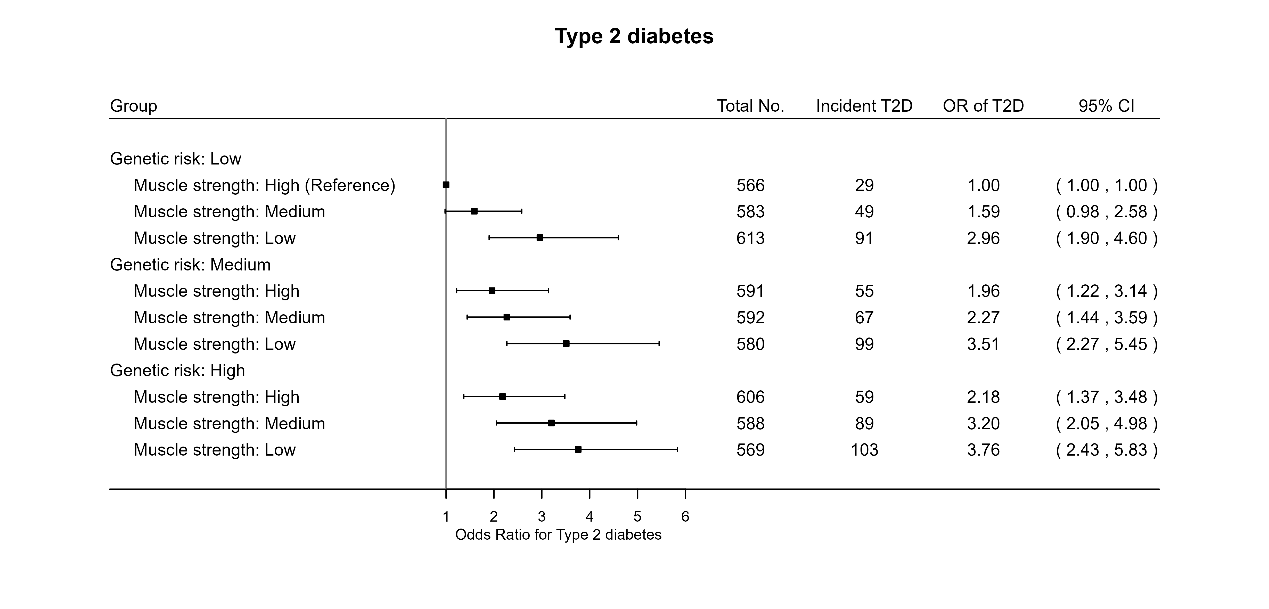


Figure S6. Joint associations of muscle strength, T2D genetic risk with incident T2D.

Logistic regression models using residuals defined muscle strength categories.

Models were adjusted for age, sex, smoking status (never, previous, current), employment (unemployed, employed), Townsend Deprivation Index, alcohol consumption (never, previous, currently <3 times/week, currently >=3 times/week), dietary factors (red meat consumption, vegetable intake, fruit intake, salt intake, fish intake), and moderate to vigorous physical activity (min/d), and the first 20 principal components of genetic variant. Abbreviations: Odds, odds ratio. T2D, type 2 diabetes.
